# The Case of Morphine Poisoning

**Published:** 1921-01-29

**Authors:** 


### THE CASE OF MORPHINE POISONING.

One of the best-remembered sights of an old-time general hospital casualty department was the opium-poisoning case—now generally a morphine-poisoning one. The dishevelled, cyanosed, and stertorous patient, after preliminary use of the stomach pump, with lavage, was propelled up and down a ward by the night porter and a dresser or two, with loud adjurations to "buck up." At intervals his skin was flipped with wet towels, and artificial respiration carried out. Then recommenced the stamping and pounding of six pairs of feet to and fro in an inner passage or emergency ward, making the night hideous and expending any amount of human energy; not very seldom all in vain. If Brauer (*Brauer's Beiträge zur Klinik der Tuberkulose*, October 1920) is to be believed, he has added another method to our more modern means. It was suggested to him by a desperate case in which a woman of forty gave herself subcutaneously .003 gram scopolamin and more than half a gram of morphine. The sound of stertorous breathing led to her bedroom door being broken in some hours later. The patient was quite unconscious and deeply cyanosed. All the usual measures failed of apparent effect, and she was only kept alive by continuous artificial respiration. Then, with signs of pneumonia in the lower pulmonary lobes, and the marks where the skin had been stimulated taking on a *post-mortem* appearance, tracheotomy was done and a tube introduced into the trachea near the bifurcation, connected with an oxygen cylinder. In less than five minutes the cyanosis improved; soon natural breathing recommenced; and after some time skin applications produced a return of consciousness. Oxygen was continued, however, for twelve hours more, and until the aspiration pneumonia resolved, six days later, the tube was not finally withdrawn. The patient went out of hospital quite well.

A second case, where nearly two grams of morphine had been taken by the mouth in coffee overnight, and the patient not discovered until next morning, showed great temporary improvement (particularly as regards cyanosis) to the above measure, but died after an injection of atropin, a mode of treatment which Brauer, following Lenhartz, is inclined to regard as dangerous. Of two further, and similarly unhopeful, cases Brauer delayed the fatal result in one and cured the other. He found that the opening of the trachea facilitated the removal of masses of tracheal mucus that were mechanically hindering the already laboured respiration. Directing the current of oxygen gas right down to the bifurcation, or even into a main bronchus, had the effect of causing cough and thus clearing the respiratory tract. He regards the rationale of this measure as relief of carbonic acid poisoning, which is the cause of death in these cases more than the specific effect on the respiratory centre of the narcotic. The oxygen ventilation may be safely continued for many hours.

